# Oxidative Stress Induced by High Salt Diet—Possible Implications for Development and Clinical Manifestation of Cutaneous Inflammation and Endothelial Dysfunction in *Psoriasis vulgaris*

**DOI:** 10.3390/antiox11071269

**Published:** 2022-06-27

**Authors:** Ivana Krajina, Ana Stupin, Marija Šola, Martina Mihalj

**Affiliations:** 1 Department of Dermatology and Venereology, Osijek University Hospital, J. Huttlera 4, HR-31000 Osijek, Croatia; ivkrajina@mefos.hr; 2 Faculty of Medicine, Josip Juraj Strossmayer University of Osijek, J. Huttlera 4, HR-31000 Osijek, Croatia; 3 Scientific Center of Excellence for Personalized Health Care, Josip Juraj Strossmayer University of Osijek, Trg Svetog Trojstva 3, HR-31000 Osijek, Croatia; ana.stupin@mefos.hr; 4 Institute and Department of Physiology and Immunology, Faculty of Medicine, Josip Juraj Strossmayer University of Osijek, J. Huttlera 4, HR-31000 Osijek, Croatia

**Keywords:** psoriasis, oxidative stress, sodium chloride, T helper 17 cells, biological drugs, endothelium, vascular

## Abstract

Although oxidative stress is recognized as an important effector mechanism of the immune system, uncontrolled formation of reactive oxygen and nitrogen species promotes excessive tissue damage and leads to disease development. In view of this, increased dietary salt intake has been found to damage redox systems in the vessel wall, resulting in endothelial dysfunction associated with NO uncoupling, inflammation, vascular wall remodeling and, eventually, atherosclerosis. Several studies have reported increased systemic oxidative stress accompanied by reduced antioxidant capacity following a high salt diet. In addition, vigorous ionic effects on the immune mechanisms, such as (trans)differentiation of T lymphocytes are emerging, which together with the evidence of NaCl accumulation in certain tissues warrants a re-examination of the data derived from in vitro research, in which the ionic influence was excluded. *Psoriasis vulgaris* (PV), as a primarily Th17-driven inflammatory skin disease with proven inflammation-induced accumulation of sodium chloride in the skin, merits our interest in the role of oxidative stress in the pathogenesis of PV, as well as in the possible beneficial effects that could be achieved through modulation of dietary salt intake and antioxidant supplementation.

## 1. Introduction

Psoriasis is a chronic, self-perpetuating inflammatory skin disease with apparent disease-related systemic comorbidities (e.g., obesity, hypertension, hyperlipidemia, peripheral arterial disease, psoriatic arthritis, etc.) that significantly reduces quality of life, and places a high socioeconomic burden on those affected by it [1]. A specific aspect of the disease progression involves substantial psychological disability, with up to 20% of those affected reporting symptoms of depression, and some exhibiting suicidal ideation extending to suicidal behavior [2,3]. 

Psoriasis has a relatively high incidence and prevalence worldwide, with significant differences between countries, reflecting their geographical location and socioeconomic status [4]. Accordingly, the incidence of psoriasis is disproportionately higher in some North American and Western European countries, characterized by high-income, high socio-demographic index (SDI) societies. According to 2019 Global Burden of Disease study, the average age-standardized prevalence was 503.6 per 100.000 inhabitants, ranging from 300.8/100.000 in low-SDI countries to 1072.2/100.000 in high-SDI countries, with the highest incidence rates in developed Western European countries (e.g., 2503.8/100.000 in France) [5]. It is worth noting that some forms of psoriasis, including nail psoriasis and psoriatic arthritis, remain largely underdiagnosed [6] so the actual numbers might be even higher. 

In recent years, significant advances in understanding the etiopathogenesis of the disease have led to the development of better treatment options, most notably biologics targeting tumor necrosis factor (TNF)-alpha(α) or the interleukin(IL)-23/IL-17 axis [7,8]. However, different response to treatment suggests a coexistence of different psoriasis endotypes, which does not necessarily reflect the observed variability in clinical presentation. 

Psoriasis has a clear genetic background. However, only part of the disease process could be linked directly to the genes and loci identified in genome-wide association or genome-wide linkage studies [9]. This underscores the importance of environmental factors and epigenetics in the early development and maintenance of the disease. Epidemiological studies have linked stress, smoking, alcohol, infections (*Streptococcus* spp.), obesity, and some drugs (e.g., nonsteroidal anti-inflammatory drugs and lithium) to the onset of the disease [10,11,12]. 

Furthermore, psoriasis patients have significant cardiometabolic comorbidities, particularly those suffering from moderate to severe forms of psoriasis. Although there is a possibility of a common genetic background between psoriasis and hypertension, obesity, diabetes, hyperlipidemia, cardiovascular and cerebrovascular diseases, there is also evidence of the direct effects associated with psoriasis, including low-grade systemic inflammation due to the cytokine overflow and oxidative stress [1,13]. To add to the complexity, dietary intake of sodium chloride has been shown to affect oxidative balance, leading to endothelial activation and vascular inflammation [14,15]. A significant finding of a recent study was the accumulation of sodium in psoriatic skin lesions [16] which, together with previously reported sodium-induced enhanced Th17 pathogenicity [17,18,19], raises the question of environmental factors with the disease-modifying capacity.

In the present review, we have summarized the current knowledge on the role of oxidative balance and tissue sodium accumulation in the pathogenesis of psoriasis and psoriasis-related comorbidities to provide a rationale for adapting antioxidant therapy and for sodium-reducing dietary interventions in the treatment protocol of psoriasis patients.

## 2. Immunopathogenesis of *Psoriasis vulgaris*

Psoriasis is a chronic cutaneous immune–mediated inflammatory disease affecting innate and adaptive immune system mechanism. It shows features of an autoimmune disease on an auto-inflammatory background, with both mechanisms co-existing and perpetuating one another in many cases [20]. In some variants of psoriasis (e.g., pustular psoriasis), activation of the innate immune system prevails, resulting in flares of self-sustaining inflammation and tissue accumulation of leukocytes, particularly neutrophils, reflecting the autoinflammatory nature of the disease [20,21,22]. However, in most cases, the initial activation of innate immunity leads to the breaking of tolerance and persistence of autoimmunity, as evidenced by the activation of T helper (Th) 1 and Th17 arms of the adaptive immune system and the accumulation of oligoclonal T cells secreting INF-ɣ and Th17-signature cytokines in the lesional skin [23,24]. 

Initial immune events in psoriasis are triggered in response to endogenous danger signals released by keratinocytes following stress, infection or injury, of which cathelicidin, human β-defensin and S100 proteins have been most studied [25,26]. In a simplified version, these antimicrobial peptides (AMPs), along with other released cytokines and chemokines, promote activation of dendritic cells, which in turn have an inherited capacity to present autoantigens to naïve autoantigen-specific T-helper cells in the regional draining lymph nodes, leading to their activation and clonal expansion. Activated Th1 and Th17 cells acquire skin-specific homing properties and accumulate primarily in the epidermis, where they continue to release vast amounts of interferon(INF)γ, interleukin(IL)-17 and other cytokines and chemokines involved in hyperproliferation, impaired maturation of keratinocytes and chemoattraction of other immune cells, resulting in self-perpetuating inflammation. In addition, recent advances in immunobiology have led to the discovery of tissue resident cells and new subsets of innate and innate-like lymphoid cells, whose role in the initiation and chronificity of psoriasis is still being examined. Notably, activation of innate lymphoid cells (ILC) 3, invariant NK T cells and γδT cells appears to be less TCR and/or IL-23–dependent and represents an additional reservoir of IL-17, the key cytokine in the pathogenesis of chronic plaque psoriasis [27,28]. 

As already mentioned, the initiation phase of psoriasis is closely linked to the release of AMPs, also called alarmins. Significantly greater expression levels of LL-37, human β-defensins (hBDs), S100 proteins, lipocalin 2 and RNase 7 have been consistently found in psoriatic plaques compared to the skin of healthy donors [29,30]. The most prominent role in both innate immunity responses and auto-antigen-specific T cells priming has been assigned to the LL37, a 4.5 kDa C-terminal fragment of cathelicidin. LL37 is released by keratinocytes and it forms complexes with self-genetic material, which are then taken up by dendritic cells. Activation of plasmacytoid dendritic cells (pDC) is mediated via LL37-DNA and LL37-RNA complexes by toll-like receptor (TLR) 9 and TLR7, respectively, resulting in type I interferons release [30]. Type I interferons direct the maturation of myeloid dendritic cells (mDC), while there is an additional direct LL37 RNA-induced pathway of mDC activation trough TLR8. Mature mDCs migrate to regional lymph nodes and secrete tumor necrosis factor alpha (TNF-α) and interleukin(IL)-12/23, thereby promoting differentiation of Th1 and Th17 cells, respectively [10,31]. Similar innate immune sensing has been reported for human beta defensin (HBD) 2 and HBD4 [32]. In addition, LL-37 has direct effects on keratinocytes and immune cells in psoriasis, thus supporting an active role for keratinocytes in the inflammatory cascade by releasing IL-1, IL-6, TNF-α cytokine, chemokines and type I interferons [33]. Suppression of apoptosis and enhancement of epidermal barrier function in keratinocytes are also attributed to LL37 [34].

Psoriasis has a clear genetic background with proven heritable disease-associated risk within families. Monozygotic twins have a 2- to 3-fold increased risk of developing psoriasis compared to dizygotic twins [35]. Among hundreds of loci and genes associated with psoriasis, PSORS1 is the most prominent locus identified on the chromosome 6p21, in the region coding for HLA-C*06:02 [36]. This allelic variant of HLA-C encodes an MHC class 1 molecule that is important for intracellular antigen presentation and immune surveillance, and is most likely involved in the presentation of autoantigens like LL37, melanocyte-associated protein ADAMTSL5 and human keratin 17 [7]. In parallel, LL37-specific peripheral blood CD4 and/or CD8 T cells were identified in two-thirds of patients with moderate-to-severe plaque psoriasis [37]. Upon stimulation with LL37, peripheral blood T cells produced IFN-γ, IL-21, IL-22 and IL-17, and showed an effector cell and skin-homing phenotype. Expansion of LL37-specific T cells correlated positively to disease severity, and was restricted to certain HLA haplotypes, including HLA-DR alleles DR1, DR4, DR11 and HLA-Cw6*02. These findings, along with the earliest reports of oligoclonal expansion of T cells in psoriatic lesions, unequivocally imply that there is an autoimmune component to the disease [38,39,40]. 

In response to stress, infection or injury, keratinocytes secrete antimicrobial peptides (LL37, S100, human β-defensin), cytokines and chemokines that promote activation of dendritic cells, which in turn have an inherited capacity to present autoantigens to naïve autoantigen-specific T-helper cells in draining lymph nodes. Activated Th1 and Th17 cells expand their colonies, gain skin-specific homing properties and accumulate primarily in the epidermis, where they continue to release large amounts of interferon(INF)γ, interleukin(IL)-17 and other cytokines. Tissue resident cells and new subsets of innate and innate-like lymphoid cells represent an additional reservoir of IL-17. Chemokines and cytokines released by keratinocytes and Th lymphocytes attract neutrophils to the skin, where they contribute to the inflammation and oxidative stress. ILC3, innate lymphoid cell 3; MAIT, mucosa-associated invariant T cell; pDC, plasmacytoid dendritic cell; mDC, myeloid dendritic cell; Treg, regulatory T cell; Th, T helper cell; INF, interferon; LL37, antimicrobial cathelicidin peptide LL37; HBD, human beta defensin; S100, calcium-binding protein S100; IL, interleukin; ADAMTSL5, A disintegrin-like and metalloprotease domain containing thrombospondin type 1 motif-like 5; γδT, gamma delta T cells. 

**Figure 1:**
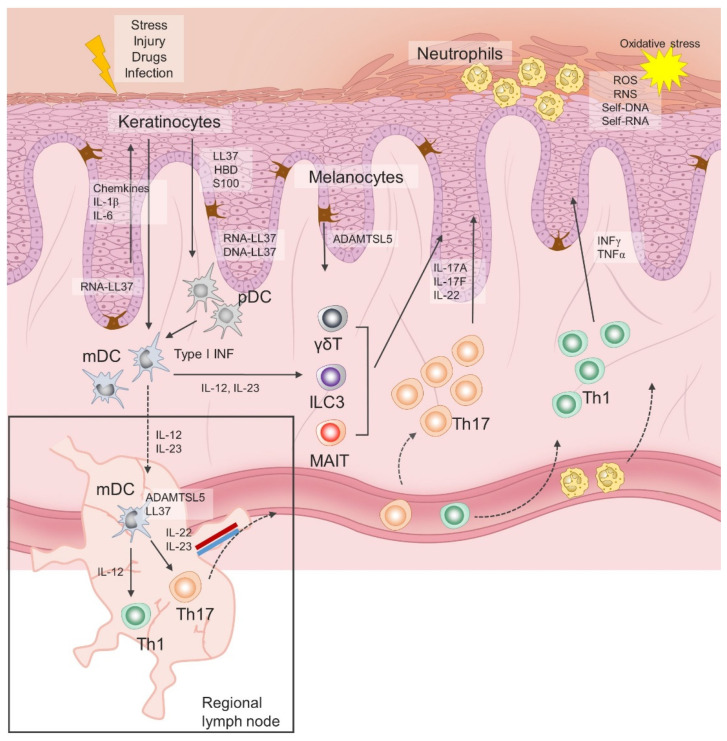
Immunopathogenesis of chronic plaque psoriasis.

Finally, disease-specific cellular and molecular events in psoriasis are directly linked to the TNF–IL-23–Th17 inflammatory pathway [41]. The IL-17 cytokine family consists of six members (IL-17A, IL-17B, IL-17C, IL-17D, IL-17E and IL17F), with IL-17A and IL-17F being primarily associated with clinically relevant signaling in psoriasis, acting through the same receptor, but with varying potencies [42]. The biological effects of IL-17A are greater than those exerted by IL-17F, while the IL-17A/IL-17F heterodimer has an intermediate effect. Binding of IL-17A to its receptor complex, composed of two IL-17RA subunits and one IL-17RC subunit, leads to recruitment of the ACT1 adaptor protein and downstream activation of a series of intracellular kinases, including I-kappa B kinase (IKK), p38 MAPK, extracellular signal-regulated kinase (ERK), TGF-beta-activated kinase 1 (TAK1), and glycogen synthase kinase 3 beta (GSK-3 beta) [43]. These kinases engage NFB, AP-1, and C/EBP transcription factors to produce pro-inflammatory cytokines, chemokines, and antimicrobial peptides. Illustration of the immune events related to psoriasis is given in Figure 1.

## 3. Increased Dietary Salt Intake Impairs Redox System Mechanism, Leading to Increased Oxidative Stress—Implications for Endothelial Dysfunction

Endothelial dysfunction is a reversible state of altered response of the endothelium to mechanical or biochemical influences, which is known to precede the process of atherosclerotic plaque formation [44,45,46]. In addition, endothelial cell activation, mechanistically closely linked to endothelial dysfunction, is characterized by an upregulated expression of intracellular, vascular, and leukocyte adhesion molecules on the surface of endothelial cells (ICAM-1, ICAM-3, VCAM-1, E-selectin) [46,47,48,49]. It has been well established that endothelial dysfunction occurs in normotensive and hypertensive individuals with known salt sensitivity, i.e., with blood pressure elevation in response to increased dietary salt intake [50,51]. In fact, it has been observed that short-term high-salt (HS) dietary intake can result in damage of endothelial function independently of blood pressure values and one’s salt sensitivity, and that oxidative stress might play a major role in the occurrence of endothelial dysfunction in high-sodium conditions [52,53]. Oxidative stress is characterized by the loss of balance between reactive oxygen species (ROS) and the antioxidative defense mechanisms [54,55]. When maintained at homeostatic levels, ROS participate in numerous physiological mechanisms, having the role of second messengers, and being a contributing factor in innate and adaptive immune defense mechanisms [56,57], which are crucial for maintaining health. However, in terms of excessive ROS production and/or decreased antioxidant activity, this redox imbalance can lead to various pathophysiological conditions, including the development of cardiovascular diseases [58,59].

A growing body of evidence from in vitro, animal, and human studies demonstrated the effects of high dietary salt loading on endothelial function independently of high blood pressure. For instance, a decline in endothelium-dependent vasodilatation in response to acetylcholine was found in normotensive rats on a high-sodium diet compared to the rats on low-salt (LS) diet, indicating that decreased endogenous NO bioavailability leads to diminished vascular dilatation. This was further supported by the finding of preserved vascular responsiveness upon the addition of NO donor sodium nitroprusside (SNP). Interestingly, this effect of HS diet was abrogated in the presence of ROS scavengers, superoxide dismutase (SOD), SOD mimetic Tempol, which is characterized by higher membrane permeability than SOD itself, and catalase [60,61]. It has therefore been suggested that HS dietary intake might cause an imbalance in redox equilibrium and increase ROS production, particularly escalating superoxide (O_2_^●−^) levels, originating both from the deterioration of the antioxidative defense apparatus and the enhanced activity of enzymes generating ROS [52,53]. 

In fact, it seems that O_2_^●−^ is the main ROS in HS-induced oxidative stress [62,63], being generated mainly by NADPH oxidase [60,63], xanthine oxidase [60,63] and uncoupled eNOS [62]. Namely, Zhu et al. found that inhibition of NADPH oxidase and xanthine oxidase leads to diminished O_2_^●−^ production and ameliorates the effect of HS diet on vascular function in a rodent model [63]. Furthermore, eNOS, the enzyme normally responsible for NO production, produces O_2_^●−^ instead of NO in the absence of the eNOS cofactor tetrahydrobiopterin (BH4) or its substrate L-arginine [64]. ROS, especially peroxynitrite (ONOO^−^), produced by oxidation of NO with O_2_^●−^, causes further oxidation of the cofactor BH4 and leads to the uncoupling of eNOS, which then produces O_2_^●−^, resulting in a vicious prooxidant cycle [58,65]. These mechanisms represent a major path of NO bioavailability reduction in the presence of O_2_^●−^, as well as a possible additional source of ROS during HS diet [65,66]. The potential role of eNOS uncoupling in HS setting is supported by the in vitro finding that the addition of BH4 to a cell culture of human umbilical vein endothelial cells (HUVECs), previously treated with high sodium, abolishes the effect of sodium on the endothelium [67]. Notably, it has been found that in rats on HS diet, the mRNA expression of inducible NOS (iNOS) was decreased in the middle cerebral artery [68]. It should be noted that in rodent studies, a significantly lowered expression of cytoplasmic copper (Cu)/zinc (Zn) SOD, as well as mitochondrial manganese (Mn) SOD, has been found in cerebral resistance arteries in Sprague-Dayley rats fed a HS diet [69]. Given that SOD is a metalloenzyme responsible for converting O_2_^●−^ to hydrogen peroxide (H_2_O_2_), which is then further reduced to H_2_O by catalase or glutathione peroxidase, such downregulation of SOD contributes to elevated O_2_^●−^ levels in settings of increased dietary salt intake [70]. Unlike SOD levels, catalase levels in vessels of animals fed a HS and normal salt (NS) diet have been found to be similar [70]. As already mentioned, addition of SOD or Tempol leads to a complete restoration of endothelial function to levels found in experimental animals with normal salt intake [66].

In addition, mitochondrial formation of ATP via oxidative phosphorylation also produces O_2_^●−^ as a by-product, which is subsequently converted to H_2_O_2_ by Mn-SOD [71,72]. Researchers previously suggested that the increased O_2_^●−^ level in a HS diet could be partly caused by increased ATP formation, compensating for the increased utilization of ATP through enhanced Na^+^/K^+^ ATPase activity [67,73,74,75]. Furthermore, the reduced Mn-SOD expression found in vessels of experimental animals fed a HS diet [69] could contribute to insufficient ROS elimination and possibly represent another mechanism for the accumulation of O_2_^●−^ originating from mitochondrial oxidative metabolism. 

Even though oxidative stress in HS diet primarily affects vascular homeostasis through the reduction of NO bioavailability, as demonstrated previously, it also may affect alternative sites in the cell, such as nucleic acids, proteins and lipids, and consequently lead to a change in several biochemical and physical processes [76]. For example, HS dietary intake seems to impair Ca^2+^ signaling pathways, as demonstrated in endothelial cells of rat aorta, where a reduction of the [Ca^2+^]_i_ amplitude has been observed in response to the addition of muscarinic receptor agonist methacholine, as well as histamine [66]. Interestingly, the acute ROS scavenging effect by adding the SOD mimic Tempol into the tissue bath did not decrease the effect of the HS diet on the reduced [Ca^2+^]_i_ response, suggesting that the impairment of Ca^2+^ signaling may not be a direct effect of O_2_^●−^. Conversely, chronic exposure to Tempol added to the drinking water before and during HS feeding increased the methacholine-induced amplitude of [Ca^2+^]_i_ in HS rats. Zhu et al. suggested that the possible explanation for this difference in acute and chronic Tempol exposure on the Ca^2+^ signaling pathways in HS diet might be due to the damage caused by the continuously elevated O_2_^●−^ to G protein/receptor-mediated signaling in endothelial cells in the aortas of rats fed a HS diet [66]. Given that multiple recent studies have shown that ROS induces an increase of [Ca^2+^]_i_ in endothelial cells, an effect attenuated by the addition of ROS scavengers, the mechanisms of HS dietary load on this attenuated amplitude of the [Ca^2+^]_i_ increase in the rat aorta have yet to be elucidated [77,78]. 

Furthermore, short-term HS diet in young healthy individuals has been linked to elevated levels of 8-iso-prostaglandin F2α (8-iso-PGF2α), a biomarker for oxidative stress, which is formed through non-enzymatic peroxidation of polyunsaturated fatty acids (PUFA), such as arachidonic acid [53,79]. Importantly, 8-iso-PGF2α acts via thromboxane prostanoid (TP) receptor activation, inducing subsequent changes in regulation of endothelial function while acting as a vasoconstrictor in multiple vascular beds, as well as inducing platelet activation and the interactions between leukocytes and endothelial cells [77,80,81,82,83], all of which may contribute to the impaired vascular health in HS diet. The fact that the addition of ascorbic acid and tocopherol prevented the increase of 8-iso-PGF2α in young healthy individuals indicates the importance of the suppression of antioxidative defense under HS dietary conditions in terms of impaired vascular health [53]. 

In an in vitro study using bifurcating flow-through HUVEC (human umbilical vein endothelial cells) cell culture mimicking arterial bifurcation, it was shown that high sodium conditions greatly increase endothelial cell susceptibility to the effects of TNF-α in conditions of non-uniform shear stress and that this effect of HS is concentration-dependent. Thus, incubation with increased sodium concentrations in vitro induced both increased expression of VCAM-1 and E-selectin and led to greater monocyte adhesion to endothelial cells in the model with non-uniform shear stress [67]. However, this HS-reinforced endothelial activation effected by TNF-α was prevented by laminar flow conditions and even somewhat conversed in static flow conditions, meaning that these sodium-induced changes in the endothelium depend on shear stress pattern [67,84]. In addition, the effect of HS concentrations to endothelial activation under non-uniform shear stress was prevented in the absence of TNF-α [67]. These findings could be of particular interest with regard to diseases such as psoriasis, in which the expression of TNF-α is enhanced, since sodium enhances the proatherogenic effect of TNF-α on cell adhesion in the bifurcation of carotid arteries (e.g., arterial areas usually prone to atherosclerosis) in ApoE-deficient mice on HS diet compared to those fed a normal-salt diet [67]. Moreover, the increased recruitment of monocytes to the endothelium under HS conditions is reduced by the addition of Tempol to the HUVEC culture, this suggests that HS exhibits this effect via O_2_^●−^. In addition, an in vitro study on lamina propria mononuclear cells (LPMC) showed enhanced expression of TNF-α and IL-17a in the presence of increased NaCl compared to LPMC in the absence of increased NaCl concentrations. Furthermore, this increase in expression of TNF-α and IL-17a was prevented by the addition of SB202190, a p38/MAPK inhibitor, leading to the conclusion that p38/MAPK has a role in the sodium-induced increased expression of TNF-α and IL-17a in LPMC [85]. Still, it remains unclear if this effect of HS could affect vascular health. 

## 4. Role of Oxidative Stress in Immunopathogenesis of *Psoriasis vulgaris*

As previously mentioned, psoriasis is a disease with genetic heredity, which still has not been fully explained. However, numerous exogenic trigger factors for psoriasis, well-known for their prooxidative influence, have been recognized, e.g., cigarette smoking, consumption of alcoholic beverages and narcotic drugs, physical and psychological stress, and infections and physical injuries [86]. Psoriasis is accompanied by enhanced ROS production and a disturbed redox equilibrium [87]. In addition to the external prooxidative influences, ROS originating from endogenous sources largely contribute to oxidative stress in psoriasis [88,89]. It has been assumed that Th1 and Th17, the two dominant T-helper cell subsets involved in the immunopathogenesis of psoriasis, can lead to ROS production via NADPH oxidase, iNOS and MPO [88,89,90].

In a study on primary human keratinocytes, it was shown that TNF-α, a prominent cytokine in the pathogenesis of psoriasis, induces H_2_O_2_ production in vitro and that this effect is dose-dependent [91], which is consistent with studies on other human cell types [92,93]. Correspondingly, increased concentrations of H_2_O_2_, O_2_^●−^ and NO have been reported in the skin of patients with psoriasis [87]. In addition, ICAM-1 overexpression was found in psoriatic keratinocytes (PK), and this expression was further increased by the addition of T lymphocytes to PK in vitro [94,95]. In contrast, the addition of lymphocytes to healthy keratinocytes (HK) did not significantly affect the expression of ICAM-1. Consequently, this increased expression of ICAM-1 seems to play an important role in the increased mobilization of neutrophils to the psoriatic plaque and their adhesion to PK. Along with these observations, results obtained from an in vitro study demonstrated that PK increased the neutrophilic production of O_2_^●−^ superiorly to HK [96]. 

It is now widely recognized that enhanced lipid peroxidation, occurring in the setting of excessive ROS levels, plays a role in the pathogenesis of psoriasis [79,97]. Lipid peroxidation is an oxidative chain process in which prooxidant ROS molecules, such as hydroperoxyl radical (HO^∙2^) and hydroxyl radical (HO^∙^), react with lipids having carbon-carbon (C-C) double bonds, e.g., PUFA [97,98]. The primary lipid peroxidation products, lipid hydroperoxides (LOOH), along with many other secondary aldehyde products, such as malondialdehyde (MDA), arise from this process [97,99,100,101], and their levels have been studied in psoriasis patients [102]. In fact, many researchers have decided to quantify oxidative stress in psoriasis by measuring LOOH, MDA, oxidized low-density lipoprotein (oxLDL) or thiobarbituric acid reactive substance (TBARS) [103], linked to the lipoperoxidation process. In recent years, another lipid peroxidation product of arachidonic acid (AA), prostaglandin F2α (8-iso-PGF2α), has emerged as a particularly sensitive and specific biomarker for oxidative stress [104]. It has been shown that MDA plasma levels are higher in patients suffering from psoriasis than in healthy controls and that the level of MDA is elevated in lesional psoriatic skin when compared to non-lesional skin [105], even though there is some evidence to the contrary [106]. Furthermore, a positive correlation has been found between MDA levels and Psoriasis Area and Severity Index (PASI) [105,107,108], along with the correlation with disease duration [107]. Moreover, MDA levels were higher in the remission phase of the disease in patients whose mean PASI was >6, suggesting that oxidative stress in patients with moderate to severe psoriasis is still present at the beginning of the remission phase [109]. Also, MDA serum levels in psoriasis correlated significantly with the serum levels of vascular adhesion protein-1 (VAP-1), an adhesion molecule included in the process of leukocyte migration to the inflammation site [110]. In addition, when it comes to high sodium dietary loading, an animal study found significantly higher MDA levels in the testicular tissue of rats fed a HS diet [111]. 

Furthermore, in patients with psoriasis, higher levels of ox-LDL have been detected in lesional skin than in non-lesional skin [112]. Another study found that 8-iso-PGF2α is elevated in serum and in fibroblasts from psoriatic plaques compared to healthy controls. In addition, psoriasis patients with severe psoriasis had higher levels of 8-iso-PGF2α than patients with mild or moderate psoriasis, and PASI correlated positively with the levels of 8-iso-PGF2α [113]. Elevated levels of AA, a PUFA which is a marked substrate for the formation of MDA via lipid peroxidation, were found in the erythrocyte membrane in patients with psoriasis [114]. Similarly, the MDA content in erythrocytes of those patients was increased as well [115]. 

Furthermore, ROS can also influence cellular signal transduction pathways, also known to play an important role in the pathogenesis of psoriasis, such as mitogen-activated protein kinase/activator protein 1 (MAPK/AP1), nuclear factor κB (NF-κB) and Janus kinase-signal transducers and activators of transcription (JAK/STAT) [87,116,117,118,119]. It appears that Th1 and Th17 lead to increased ROS levels, which causes, via these redox-sensitive pathways, a self-reinforcing loop due to their ability to upregulate the expression of pro-inflammatory cytokines important for the development of psoriasis [88,120]. 

In addition, levels of sirtuin 1 (SIRT1), a NAD^+^-dependent protein deacetylase that removes acetyl groups from various proteins and also acts as a transcription factor, may be important in psoriasis. As SIRT1 endorses keratinocyte differentiation and is able to inhibit keratinocyte proliferation [121,122], the decreased expression and activity of SIRT1 in fibroblasts of skin samples from psoriatic plaques suggest that SIRT1 has an important role in psoriasis [113,123,124]. In particular, evidence for the role of SIRT1 in restoring redox balance in psoriasis was obtained from an in vitro study of psoriatic fibroblasts in which the levels of 8-iso-PGF2α and intracellular ROS were significantly reduced after the addition of SRT1720, a selective activator of SIRT1 [113]. Dietary salt intake was observed to have an effect on SIRT1 levels in various tissues of a rodent model. Specifically, after 7 days of LS diet, the expression of SIRT1 was increased in the kidney and in all the tested extrarenal tissue samples (brain, hart, muscle and fat), while it did not change significantly in rats fed a HS diet [125]. In addition, SIRT1 appears to have a protecting function against mitochondrial injury and it affects redox balance by affecting all three redox-sensitive MAPK signaling pathways [113]. 

Members of MAPKs family (Ser/Thr protein kinases), which are included in redox-sensitive pathways and play a role in cellular signaling of oxidative stress, are the extracellular signal-regulated kinase (ERK), c-Jun N-terminal kinase (JNK) and the p38 MAPK [119,126,127]. The transcription factor activated by all three MAPK pathways, AP-1, is also important in the regulation of transcription of many molecules, including cytokines involved in psoriasis pathogenesis such as TNF-α and IL-6 [119]. Becatti et al. found that the level of phosphorylated ERK, which appears to have an antiapoptotic effect, is decreased in psoriatic fibroblasts compared to fibroblasts from healthy controls, while the treatment with SRT1720 led to an increase in ERK phosphorylation and activation. These findings are supported by previous research, in which SIRT1 activation induced phosphorylation of ERK in human fibroblasts in vitro [128]. In contrast, the addition of the inhibitor of SIRT1 led to further deterioration of ERK phosphorylation [113]. However, there is some conflicting evidence as other researchers have found that the level of phosphorylated ERK1/2 is higher in affected psoriatic skin [129,130] and that the activation of ERK by ROS has been observed in a variety of human cell types [87], so further studies are necessary to clarify the interaction of ROS and ERK in the context of psoriasis. Furthermore, expression and phosphorylation of JNK was enhanced in lesional psoriatic skin and in psoriatic fibroblasts compared to non-lesional skin and healthy fibroblasts [113,130]. JNK can be activated by various stimuli, including ROS [131,132]. In an in vitro study on psoriatic fibroblasts, the addition of SIRT1 activator led to a decrease in phosphorylation of JNK, whereas the inhibition of SIRT1 conversely led to its increase, suggesting that SIRT1 inhibits JNK phosphorylation and activation [113]. This is particularly interesting as it has been shown that the inhibition of JNK with SP600125 leads to epidermal differentiation (a process impaired in psoriasis) by enhancing transcription of cornification markers, inducing stratification and causing the formation of the cornified envelope [133]. These findings may also have therapeutic implications for psoriasis management. 

In addition, the activity of p38 MAPK [129,134], is elevated both in lesional psoriatic skin [129], and in psoriatic fibroblasts [113]. This pathway is activated by various ROS, including O_2_, H_2_O_2_, NO and ONOO [135,136,137,138,139]. SIRT1 also plays a role in the phosphorylation of p38 MAPK, since the addition of SIRT1 activator caused a significant decrease in p38 MAPK phosphorylation [113]. In addition, adalimumab inhibited p38 MAPK in the affected skin of psoriasis patients prior to the clinical improvement of plaques, suggesting that modulation of p38 MAPK pathway is mechanistically involved in the effect of this biologic drug [140]. There is also a link between MAPK/AP1 and NF-κB signaling pathways via mitogen and stress activated kinase 1 (MSK1), a kinase which is activated downstream of ERK and p38 MAPK, and which causes NF-κB phosphorylation, resulting in increased NF-κB transcriptional activity, including increased AP1 expression [141,142,143].

Furthermore, ROS leads to degradation of the inhibitory protein I-κB, which, due to the attenuated inhibition, causes greater transcriptional activity of NF-κB, which then further induces the expression of pro-inflammatory cytokines involved in the pathogenesis of psoriasis [144,145,146]. The active phosphorylated form of NF-κB expression was significantly increased in lesional psoriatic skin compared to non-affected skin [147]. Some prooxidative, ROS-producing enzymes are upregulated by the NF-κB pathway, such as NADPH oxidase [148], xanthine oxidase [149], and iNOS [150,151,152,153]. 

Another redox-sensitive pathway important in psoriasis is JAK/STAT. STAT 1 and STAT3 are activated in fibroblasts in response to the addition of H_2_O_2_ [116]. JAK/STAT activation after stimulation with oxLDL is inhibited by the antioxidant treatment with vitamin E, underlining the importance of the antioxidant defense system [154]. Importantly, expression of iNOS, a known downstream gene of JAK/STAT, is also enhanced in psoriatic lesional skin compared to normal skin [155]. 

As regards the antioxidative defense mechanisms in psoriasis, evidence seems to be controversial. On the one hand, the evidence from most studies supports a weakened antioxidative status, reporting decreased SOD and CAT levels, which negatively correlate with disease severity assessed by PASI [107,156,157,158,159], implying that this further worsens the imbalance between prooxidative and antioxidative mechanisms, leading to increased oxidative stress. On the other hand, some researchers report increased activity of antioxidative enzymes and suggest that it could be due to the transcriptional regulation of SOD, CAT, and glutathione peroxidase by redox balance and due to the interaction between antioxidant responsive element (ARE) and the nuclear factor erythroid 2-related factor 2 (Nfr2) [160]. 

As discussed in the previous section, sodium can potentially cause oxidative stress through enhanced prooxidant and weakened antioxidant activity [53]. Importantly, the plasma levels of sodium are tightly regulated by osmotic mechanisms within a homeostatic range between 135 and 145 mmol/L, even in terms of high salt dietary loading, which is important for the regulation of intravascular blood volume, systolic blood pressure and osmolarity. However, it has been suggested that the excess sodium is not eliminated from the organism completely, and the excess sodium that is not excreted can accumulate in the interstitial space, leading to hypertonic sodium storage [161,162,163,164]. Results from animal studies imply that glycosaminoglycans can bind sodium in the skin, leading to higher hypertonic storage [165]. Another possible mechanism could also be countercurrent exchange with urea [166]. Maifeld et al. showed that patients with PASI > 5 had significantly increased sodium content when estimated non-invasively by ^23^Na magnetic resonance imaging (MRI) compared to healthy controls, and they subsequently reaffirmed those findings by atomic absorption spectrometry measurements on skin biopsies ex vivo. In addition, sodium content in non-lesional skin was elevated and positively correlated with PASI. Interestingly, sodium content did not differ significantly between lesional and non-lesional skin [16]. Since this study did not assess participants’ sodium dietary intake, the impact of dietary salt intake on sodium accumulation in patients with psoriasis remains unknown [167]. These findings from human studies were consistent with data obtained from experiments on both the IMQ mouse model and three IL-17A–driven psoriasis murine genetic models compared to controls [16]. There is a great need for further studies in this field to explain the mechanisms of sodium accumulation in the skin and the effect of oxidative stress, possibly associated with elevated sodium levels, on the pathogenesis of psoriasis. It has also been suggested that these findings could have therapeutic implications, since LS diet could potentially decrease sodium content in the skin of patients with psoriasis [167]. 

## 5. Evidence for NaCl-Mediated Modulation of Type-3 Inflammation—Dichotomous, Context-Dependent Effect of NaCl on the Th17 and Treg Phenotype

Naïve T helper cells have an inherited capacity to adapt their maturation process after an antigen encounter in relation to their changing environment and the nature of antigenic challenge. Their tremendous plasticity is reflected in a growing family of Th subsets, including Th1, Th2, Tfh, Th17, Th9 and Th22 etc. [168]. Classical signals for T-cell activation and differentiation include recognition of peptide-MHC complex by their cognate T-cell receptor, engagement of costimulatory receptors, and cytokine signaling. Fine-tuning of diverse polarizing cytokines and their downstream signaling have been recognized as the major force driving T lymphocytes towards their respective subset. Type 3 immunity referees to the immune responses mediated by cells that produce IL-17 family cytokines, which are excreted during infections by pyogenic extracellular bacteria at epithelial barriers, and alternatively in the case of aberrant initiation such immune responses can lead to autoimmunity [169]. The best examples of the central role of signature cytokines in an inflammatory process came from clinical setting, including patients with psoriasis who achieved clear or almost clear (disease-free) skin upon receiving IL-17 or IL-23 blocking agents [170].

Moreover, recent studies have provided evidence for divergent post-activation fates of T-cells when local conditions related to cytokine milieu have changed. For example, presence of proinflammatory cytokines, IL-6 alone or in combination with IL-1β, can drive transdifferentiation of Foxp3+ regulatory T cells into either Th17 or Treg/Th17 cells, respectively [171]. That way, regulatory T cells lose their capacity to maintain peripheral tolerance and limit immune responses, and gain proinflammatory effector functions [171]. Beyond the classic signals, increasing evidence suggests that environmental factors and metabolic changes in the surrounding microenvironment could also drive T cell (trans)differentiation and adaptation during inflammation and immune sensing. These external signals include vitamins [172,173], oxygen level [174], reactive oxygen and nitrogen species [175], local metabolites from host cells and commensal or pathogenic microorganisms [176] and, most recently, sodium chloride (NaCl) [177]. This is very important given the fact that naïve and memory T cells recirculate between the secondary lymphoid organs and the sites of inflammation, and, in so doing, change their microenvironmental conditions. Here, we focus on the evidence supporting an important role of tissue NaCl in both shaping and constraining Th17 immune responses. 

It was long postulated that NaCl concentrations in body fluids and tissues are tightly regulated by the kidneys and the endocrine system. Assessment of serum sodium concentration in animals and humans under various physiological and pathological conditions showed that it is maintained within a narrow range [178,179]. However, this is not true for the tissues where precise measurement was not possible until recently. In the tissues, NaCl rapidly dissociates into positively charged sodium ions (Na^+^) and negatively charged chloride ions (Cl^−^). Free Na^+^ ions then rapidly establish noncovalent bounds with negatively charged tissue macromolecules, such as glycosaminoglycans and hyaluronic acid, thus preventing determination of their precise tissue concentrations [177]. Advanced techniques, including neutron activation analysis and sodium (^23^Na) magnetic resonance imaging, have brought to light the fact that tissue sodium content dynamically adapts to dietary changes in salt intake and exhibits variable sodium tissue content depending on the type of tissue, sex, age, and inflammation status [180,181,182].

**Figure 2:**
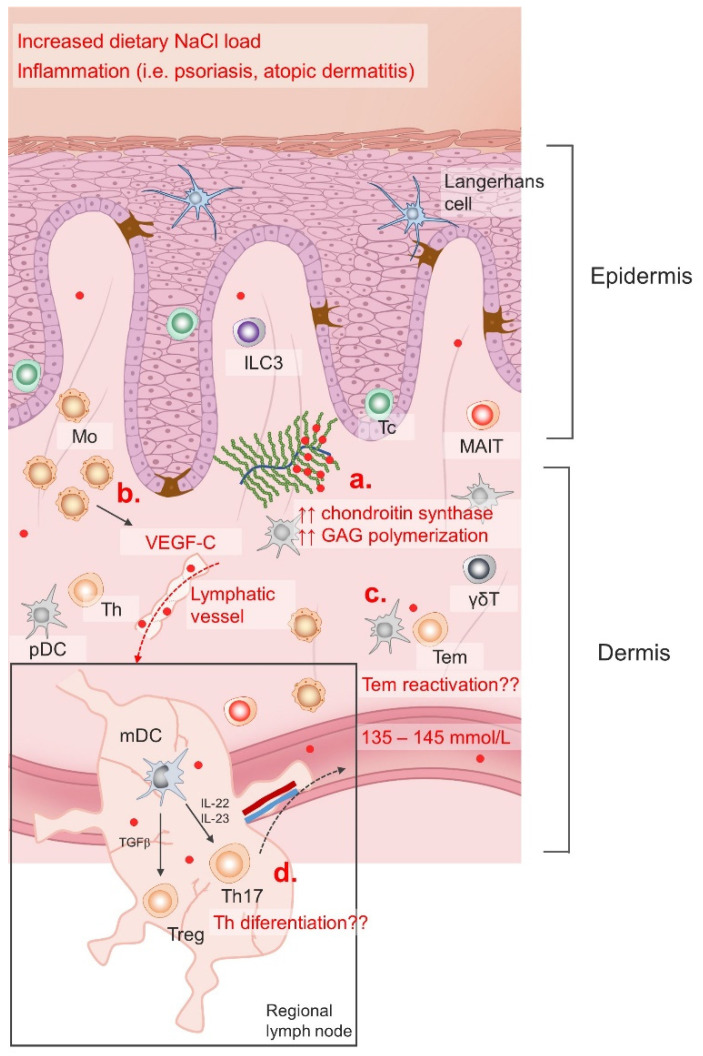
Regulation of skin NaCl storage. Serum NaCl (red dots) concentration is maintained within a narrow range (135–145 mm/L) with varying dietary intake and in different pathological conditions; however, recent data suggests that the skin NaCl content changes significantly in response to dietary load and inflammation. Increased GAG polymerization (a) enables Na+ storage, while neo(lympho)angiogenesis allows NaCl clearance in the skin induced by VEGF-C stimuli (b). Skin is an important lymphoid compartment characterized by immune surveillance cells. Increased Na+ content affects Tem reactivation (c) in the skin, as well as naïve Th lymphocytes differentiation in the regional lymph nodes (d).

Tissue particularly sensitive to dietary changes in sodium intake is the skin [181]. Furthermore, inflammatory skin conditions such as atopic dermatitis (AD), psoriasis and bacterial infections provided additional clues/mechanisms for sodium accumulation in the skin [16,161,183]. Interestingly, an increased number of chondroitin synthase transcripts was found in the skin following dietary NaCl accumulation, suggesting that the extent of glycosaminoglycan (GAG) chain polymerization may regulate sodium content in the skin and other tissues [184]. There is also evidence of an additional regulatory mechanism via modulation of lymphatic flow. Namely, the macrophages are able to sense changing concentrations of Na^+^ and produce vascular endothelial growth factor C (VEGFC) leading to lymph capillary hyperplasia/lymphangiogenesis and increased salt clearance from cutaneous deposits [185]. Illustration of the regulation of skin sodium accumulation is given in Figure 2.

Mo, monocytes; ILC3, innate lymphoid cell 3; Tc, cytotoxic T cell; MAIT, mucosa associated invariant T cell; pDC, plasmacytoid dendritic cell; mDC, myeloid dendritic cell; Treg, regulatory T cell; Th, T helper cell; Tem, effector memory T cell; GAG, glycosaminoglycans; VEGF-C, vascular endothelial growth factor C; IL, interleukin; TGFβ, transforming growth factor beta; γδT, gamma delta (γδ) T cells.

Moreover, this mechanism seems to be important for blood pressure regulation during acute salt loading, since in an experimental animal model, VEGF-C receptor blockade led to an immediate blood pressure increase [165,185]. This is further corroborated by the finding of increased sodium content in the skin of patients with hypertension [186].

In the case of psoriasis, a prototypical TNFα–Th17 disease model, recent data demonstrated increased sodium and water retention in lesional and non-lesional skin, but only in patients with a moderate to severe form of the disease (PASI > 5), and not in those with a mild form (PASI < 5). Sodium accumulation correlated to the disease severity and frequencies of peripheral blood IL-17-secreting ɣδ T cells [16]. Consistent with previous reports, the authors of the same study reported increased IL-17A production from naïve CD4 T cells primed under Th17-polarizing cytokines and high NaCl concentrations.

**Figure 3:**
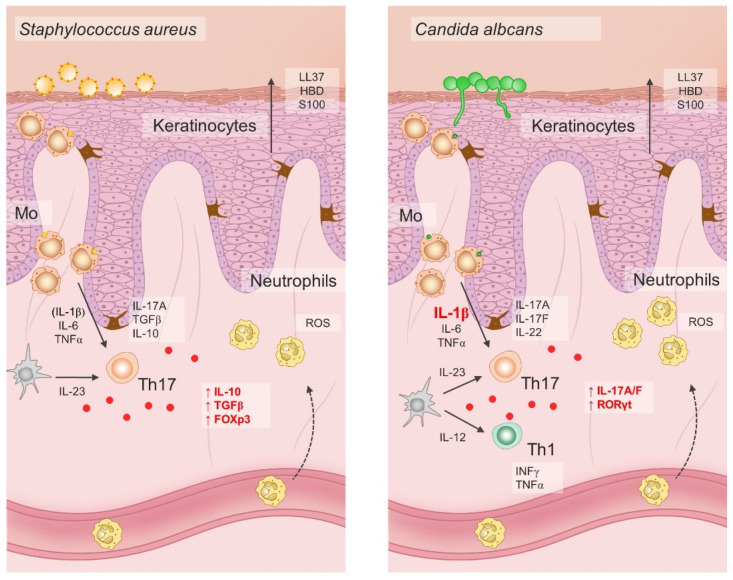
Dichotomous pathogen-specific Th17 responses under hypersaline tissue microenvironment depend on differential priming requirements for IL-1β.

Pioneering studies onionic-salt-induced effects on the Th17 cell differentiation also demonstrated increased Th cell pathogenic effector functions exerted by high NaCl [17,187]. Namely, adoptive transfer of naïve CD4 T cells primed under Th17-polarizing cytokines and increased NaCl concentrations resulted in aggravated disease symptoms of murine colitis and experimental autoimmune encephalomyelitis. Later experiments showed that the immunomodulatory effects of NaCl were not exclusive to naïve T helper cells, but that increased NaCl concentrations readily affected effector memory T cells in the same manner, even in the absence of Th17 polarizing factors [188]. Interestingly, when human effector Th17 cells were restimulated ex-vivo under hypertonic NaCl conditions in the absence of pro-inflammatory cytokines, they gained anti-inflammatory phenotype characterized by the upregulation of Foxp3, TGFβ, IL10, LAG3, ICOS, and CTLA4 transcripts [188]. Pharmacological blockade of p38 signaling abrogated NaCl-induced IL-17A and FoxP3 upregulation in effector Th17 cells, suggesting that the observed effects were mediated trough activation of p38/MAPK pathway and its downstream targets NFAT5 and SGK1 [189]. However, if the same cells were primed in a proinflammatory cytokine milieu, primarily in the presence of IL-1β and/or IL-6, their Th17 cell signature properties, such as ROR-γt, IL-22, and CCR6 expression, were preserved [188]. Furthermore, it seems that the effect was not uniform in the case of all effector Th17 cells. Rather, it depended on the cytokine conditions present during the first antigen encounter. For example, *C. albicans*-specific Th17 responses depend on the presence of IL-1β, thus such effector cells respond to hypertonic NaCl conditions by amplification of Th17 cell–associated proinflammatory effector functions [188], as illustrated in Figure 3. 

The effects of hypersaline conditions on Th17 differentiation and phenotype depend on the presence of pro-inflammatory cytokines such as IL-1β and IL-6. In addition, specific microbes possess differential ability to induce IL-1 production in antigen-presenting cells. Hence, increased NaCl conditions may have both pro-inflammatory and anti-inflammatory effect on the Th17 cells in response to invading microbes. For example, hypersaline microenvironment has been shown to increase the anti-inflammatory functions of S. aureus-specific Th17 lymphocytes, while it promotes the proinflammatory functions of C. albicans-specific Th17 cells; LL37, cathelicidin antimicrobial peptide LL37; HBD, human beta defensin; S100, calcium-binding protein S100; IL, interleukin; TGF-β, transforming growth factor β; FOXp3, forkhead box P3; ROS, reactive oxygen species; IFN-γ, interferon γ; TNF-α, tumor necrosis factor α; Th17, T helper 17 cells; Th1, T helper 1 cells; RORγt, retinoic-acid-receptor-related orphan nuclear receptor γ; Mo, monocytes.

Taken together, the effects of hypersaline tissue microenvironments on the Th17 cells are divergent, both pro-inflammatory and anti-inflammatory, and dependent on the context, primarily on the presence of pro-inflammatory cytokines like IL-1β and IL-6. These findings have possible implications for therapeutic strategies in the treatment of chronic autoimmune diseases. Targeted abrogation of the IL-1 signaling pathway by IL-1- or IL-1R-blocking agents could induce anti-inflammatory Th17 responses in high-NaCl tissue microenvironments. Alternatively, tissue salt accumulation reduced through modification of dietary habits may possibly promote anti-inflammatory Th17 effector functions in patients with psoriasis and other autoimmune diseases.

## 6. Deleterious Effects of Increased Systemic Oxidative Stress and Low-Grade Inflammation on Endothelial Function in Psoriasis Patients

Since the same pro-inflammatory IL23/Th-17 axis is known to promote both psoriasis and cardiovascular diseases (CVD), psoriasis could be considered as an independent risk factor for development of CVDs (e.g., atherosclerosis, myocardial infarction) and increased cardiovascular mortality [190,191]. 

**Figure 4:**
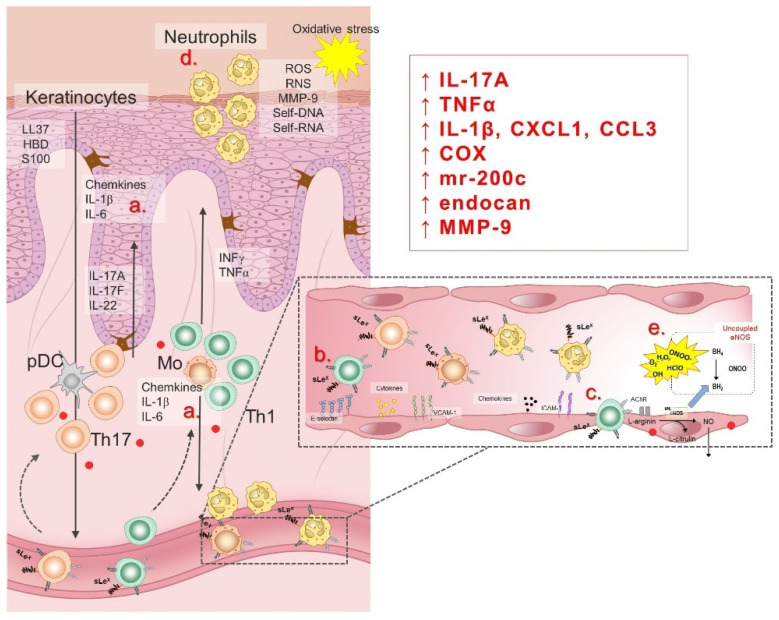
Pathogenesis of endothelial dysfunction in psoriasis patients is related to inflammation and oxidative stress. In response to the hypersaline microenvironment, keratinocytes and lymphocytes (a) secret pro-inflammatory cytokines (i.e., interleukin (IL)-1β, IL-6, TNFα) and chemokines, leading to endothelial activation characterized by the cell adhesion molecules (CAMs) upregulation (b). In response to the chemokines and interactions with CAMs, peripheral leukocytes transmigrate trough the vessel wall to the sites of inflammation (c). Neutrophils are predominant cell type in leukocyte infiltrate of the psoriatic skin, responsible for generation of reactive oxygen (ROS) and nitrogen (RNS) species (d). An additional mechanism of oxidative stress during increased dietary salt intake involves RAS suppression and eNOS uncoupling (e). LL37, cathelicidin antimicrobial peptide LL37; HBD, human beta defensin; S100, calcium-binding protein S100; IL, interleukin; ROS, reactive oxygen species; RNS, reactive nitrogen species; MMP-9, matrix metalloproteinase 9; self-DNA, self-deoxyribonucleic acid; self-RNA, self-ribonucleic acid; IFN-γ, interferon γ; TNF-α, tumor necrosis factor α; pDC, plasmacytoid dendritic cell; Th17, T helper 17 cells; Th1, T helper 1 cells; sLe^x^, Sialyl Lewis X; CXCL1, C-X-C motif chemokine ligand 1; CCL3, C-C motif chemokine ligand 3; COX, cyclooxygenase; mr-200c, microRNA-200c; endocan, endothelial cell specific molecule-1; ICAM-1, intercellular adhesion molecule 1; VCAM-1, vascular cell adhesion molecule 1; AChR, acetylcholine receptor; eNOS, endothelial nitric oxide synthase; NO, nitric oxide; BH4, tetrahydrobiopterin; ONOO, peroxynitrite; H_2_O_2_, hydrogen peroxide; O_2_^−^, superoxide; OH, hydroxide; HClO, hypochlorous acid.

Endothelial dysfunction (ED), an early sign of CVDs, encompasses a number of pathophysiological conditions, ranging from initial localized mechanical injury to inappropriate, persistent, global endothelial activation. ED is characterized by vasoconstriction, pro-thrombotic and pro-inflammatory phenotype [192,193], and it commonly refers to reduced production and/or bioavailability of vasodilator NO, as well as an imbalance in the relative contribution of endothelium-derived relaxing and contracting metabolites, resulting in impaired vascular relaxation mechanisms [194]. In addition to damaged endothelium-dependent vasodilation, the pathophysiological consequences of ED development are: (1) abnormal vascular reactivity and vascular spasm; (2) increased endothelial permeability to macromolecules; (3) increased expression of soluble cell adhesion molecules (sCAMs); (4) recruitment and accumulation of monocytes/macrophages in blood vessels intima; (5) reduced regeneration of endothelial cells and increased proliferation/migration of smooth muscle cells; and (6) hemostatic equilibrium disorder [195,196,197]. Pathophysiological factors known to contribute to the development of ED are: increased oxidative stress due to formation of ROS, activation of cytokines in inflammatory processes, glycosylation of metabolites involved in diabetes, aging, smoking and hypertension, chronic hypercholesterolemia and/or elevated plasma LDL concentration and its accumulation in the blood vessel wall, as well as chronic systemic infection with bacteria, viruses or other pathogens [195,196,197,198,199,200]. Considering that psoriasis is a chronic inflammatory disease accompanied by increased levels of oxidative stress, ED is to be expected and certainly associated with high cardiovascular risk in psoriasis patients. Illustration of pathogenetic mechanisms of ED in psoriasis is given in Figure 4.

Over the last 40 years, most studies on endothelial function in psoriasis have reported impaired macrovascular endothelial function in psoriasis patients, as assessed by flow mediated dilation (FMD) of brachial artery [201,202,203,204,205], pulse wave velocity (PWV), peripheral arterial tonometry (PAT) [206], carotid intima-media thickness (cIMT) [207], or the level of aortic stiffness [206]. Only few studies have reported that psoriasis patients have unaffected macrovascular endothelial function [208]. Moreover, several studies have reported that macrovascular endothelial dysfunction in psoriasis patients was directly associated with psoriatic disease activity, assessed by the Psoriasis Area and Severity Index (PASI), the extent of inflammation (hsCRP), and such patients’ cardiovascular risk. Table 1 summarizes the data from these studies.

Surprisingly, there is little data on microvascular endothelial function in psoriasis patients, particularly data investigating the cutaneous microcirculation, whose impairment may play an important role in pathophysiology of cardiovascular and metabolic diseases, especially diseases specifically affecting the skin [209], as is the case with psoriasis. One study reported impairment of skin microvascular endothelial function in psoriasis patients, assessed by measuring the index of skin blood flow during local heating (42 °C) using laser-Doppler flowmetry (LDF) [210]. In addition, the same study reported unimproved NO-dependent microvascular dilation following microdialysis of L-ascorbate, suggesting that impaired endothelium-dependent vasodilation in psoriasis occurred independently from increased oxidative stress. 

Few studies examined available biomarkers of endothelial function in psoriasis patients, and the results obtained were in line with the findings of functional vascular studies. Abdou et al. reported that psoriatic skin exhibited over-expression of endocan (a proteoglycan expressed by endothelial cells), reflecting the ongoing endothelial activation and neovascularization [211]. Similarly, Balta et al. reported increased level of circulating endocan in psoriasis patients, which also correlated with PASI, hsCRP and cIMT [212]. In vitro studies designed to assess the mechanisms underlying endothelial dysfunction observed in psoriasis patients (e.g., inflammation, oxidative stress) provided interesting results. The finding that neutrophils infiltration of psoriatic skin lesions induced vascular remodeling, potentially mediated by matrix-metalloproteinase 9 (MMP-9) release, advanced the knowledge on the role of neutrophils in the pathogenesis of psoriasis [213]. A study using direct brachial venous endothelial sampling demonstrated that psoriasis patients exhibit impaired endothelial cell vascular health manifested as inflammatory transcript upregulation (e.g., IL-1β, VCAM-1, IL-8, CXCL1, ICAM-1, COX-2, and CCL3), which could involve activated platelets, as suggested by Garshick et al. [214]. Magenta et al. reported upregulation of miR-200c in skin lesions and plasma of psoriasis patients, known to be induced by reactive oxygen species (ROS) and responsible for apoptosis, senescence, ROS increase, and nitric oxide decrease, causing endothelial dysfunction. Their findings suggested that such upregulation plays a role in ROS increase and inflammation associated with CV risk in psoriasis [215]. 

At present, there is limited systematic data evaluating the potential clinical implications of the above findings and investigating how biologic therapy may affect endothelial function and CV risk in psoriasis patients. Several studies have shown that TNF-alpha inhibitors improve endothelial function in psoriasis (e.g., improved aortic stiffness [216], decreased sCAMs [217]). For example, a Spanish experimental study reported decreased peripheral microvascular resistance in psoriasis patients (nailfold vessel resistance index) following a 52-week TNF-alpha inhibitor administration (adalimumab) [218]. Similarly, a 6-month prospective study reported improved FMD of brachial artery and arterial stiffness parameters in patients with psoriasis [219]. CARIMA study (Evaluation of Cardiovascular Risk Markers in Psoriasis Patients Treated with secukinumab) reported lower baseline FMD in psoriasis patients than in healthy volunteers. In addition, administration of a fully human monoclonal antibody against IL-17A (secukinumab) for 12 weeks increased the FMD of psoriasis patients, and its administration for 52 weeks significantly increased their FMD, indicating its potential beneficial effect on CV risk by improving the endothelial function of patients with plaque psoriasis [220]. Similarly, a recent study reported that psoriasis patients had slightly impaired brachial artery FMD compared to healthy subjects, but very similar to CVD patients. TNF-α blockade treatment significantly increased low-flow-mediated constriction (l-FMC), but not FMD, suggesting that such anti-inflammatory treatment improves vascular function in psoriasis patients, mainly by altering the baseline vascular tone, but not the vascular reactivity to given stimuli [221]. In contrast, administration of infliximab decreased reactive hyperemia-peripheral arterial tonometry index (RHI) in non-responders, but remained unchanged in patients who responded to therapy, indicating that the decrease in RHI may serve as a predictor for the unfavorable effect of infliximab on psoriatic skin lesions [222].

Thus, data available to date (both molecular and functional) provide evidence on significantly impaired macrovascular endothelial function in psoriasis patients, possibly induced by the modulation of inflammatory responses (involving TNF-alpha, IL-17A) and increased levels of oxidative stress. However, unexplored endothelial function in microcirculation (especially cutaneous), as well as a more precise assessment of the role of oxidative stress in the pathogenesis of endothelial dysfunction in psoriasis patients open up new avenues for future research. 

**Table 1:** Endothelial dysfunction in psoriasis and evidence of oxidative stress and inflammation.

First Author, Year, [Ref.]	Country	Study Design	Study Groups		Psoriasis Severity/PASI Inclusion Criteria	PASI Mean ± SD/[SEM] or Median (IQR)	Disease Duration (Years, Mean ± SD or [SEM])	Systemic Antipsoriatic Therapy	Assessment Method/ Occlusion Site	Vessel, Measurement Site	Measurements	Effect on Measured Inflammation and Oxidative Stress Parameters
			Psoriasis	Controls								
**Jensen, 2011,** [208]	Denmark	Case-control Study	30	30	Mild to moderate psoriasis/PASI < 10	7.3 ± 3.8	21.3 ± 17.0	None	PAT (reactive hyperemia index, RHI; augmentation index, %)/Brachial artery (occlusion)	both index fingers	↔ RHI; ↔ AI%	↑ hsCRP (*p* = 0.011)
**Mallbris, 2008,** [223]	Sweden	Case-control Study	20	20	Severe psoriasis/PASI > 12	14.3 ± 4.8	0.4 ± 0.3	None	FMD, NMD (absolute value vessel dilatation (B2-B1); vessel dilatation as the % of baseline value, %)/Forearm cuff (occlusion)	BA, above the elbow	↔ B2-B1 ↔ %FMD ↔ %NMD	↑ hsCRP (*p* < 0.05)
**Martyn-Simmons, 2011,** [205]	United Kingdom	Prospective Cohort Study	60	117	Moderate to severe psoriasis/PASI > 10	9.15 ± [0.91]	31 ± [1.6]	Standard systemic therapy: MTX (*n* = 16, 26.7%); Acitretin (*n* = 5, 8.3%); ciclosporin (*n* = 3, 5%), fumaric acid esters (*n* = 5, 8.3%); Biologics: anti TNF-therapy (*n* = 13, 21.7%)	FMD, NMD (vessel dilatation as the % of baseline value, %)/Forearm cuff (occlusion)	BA, above the elbow	↔ %FMD ↔ %NMD FMD associated with ciclosporin (β = 0.29, *p* < 0.04)	↑ hsCRP (*p* < 0.05)
**Gisondi, 2009,** [224]	Italy	Case-control Study	39	38	Moderate to severe psoriasis/PASI > 10	12.4 ± 4.7	14.8 ± 12.7	None (at least 2 months before inclusion)	cfPWV; no occlusion site crPWV(m/s); no occlusion site	cfPWV—sensor on CA and FA; crPWV—sensors on CA and RA	↑ cfPWV (*p* = 0.001); positive correlation with disease duration (*p* = 0.0001), not with PASI. ↔ PWVcr	↔ CRP
**Balci, 2008,** [207]	Turkey	Case-control Study	43	43	All PASI included	6.5 ± 4.4	13.26 ± 10.55	None (*n* = 32, 74%) Standard systemic treatment: acitretin (*n* = 10, 23%) Biologics: etanercept (*n* = 1, 2.3%)	cIMT (mm), no occlusion site FMD, NMD (vessel dilatation as the % of baseline value, %)/Forearm cuff (occlusion)	cIMT—left and right CCA FMD-BA, above the elbow	↑ cIMT (*p* = 0.003) ↓ FMD% (*p* = 0.002), correlating with disease duration (β = −0.259, *p* < 0.05) ↓ NMD% (*p* = 0.013) No association with systemic therapies found.	/
**Ulusoy, 2010,** [202]	Turkey	Case-control Study	28	28	Mild to moderate/PASI 0.1–49.9	13 ± 8	4 ± 3	None	FMD, NMD (vessel dilatation as the % of baseline value, %)/3–4 cm proximal to the section of the brachial artery (occlusion)	BA, above the elbow	↓ FMD% (*p* < 0.001) ↔ NMD%	/
**Von Stebut, 2019,** [220]	Germany	Randomized Controlled Trial	151 (35 + PsA)	44	Moderate to severe/PASI > 10	A. 19.3 ± 7.9 B. 21.7 ± 10.5 C. 17.5 ± 4.2 D. 19.5 ± 6.1	A. 20.6 ± 12.7 B. 20.8 ± 13.3 C. 18.9 ± 11.7 D. 20.3 ± 11.7	A. secukinumab 300 mg from baseline to week 52 (*n* = 48) B. secukinumab 150 mg from baseline to week 52 (*n* = 54) C. placebo until week 12, then secukinumab 300 mg until week 52 (*n* = 26) D. placebo until week 12, then secukinumab 150 mg until week 52 (*n* = 23)	FMD (vessel dilatation as the % of baseline value, %)/5 cm distal to the measurement site (occlusion) PWVcf (distance/Δtime [m/s]), AI [%], no occlusion site	FMD—BA, 5–10 cm proximal to the antecubital fossa PWVcf—on CA and FA	Psoriasis patients compared to healthy controls: ↓ FMD% (at baseline), (*p* < 0.01) Group A and B compared to 3 and 4 at 12 weeks: ↔ FMD% Group A compared to baseline: ↑ FMD% (*p* < 0.002) Group B compared to baseline: ↑ FMD% (*p* = 0.0034) Group D compared to baseline: ↔ FMD%, ↔ PWVcf	A compared with C + D at week 12: ↓ S100B (mean −0.02, 95%CI −0.03 to 0.01)
**de Simone, 2011,** [201]	Italy	Case-control Study	32	31	Not specified/all PASI included	17.9 ± 10.9	12.6 ± 10.2	None (at least 3 months prior)	FMD, NMD (vessel dilatation as the % of baseline value, %)/forearm (occlusion)	Right BA, 2 to 15 cm proximal to the antecubital fossa	↓ FMD% (*p* = 0.012), no correlation found with PASI or disease duration ↔NMD%	↔ CRP ↔ ESR
**Erfan, 2005,** [225]	Turkey	Case-control Study	60	30	Moderate to severe/PASI ≥ 5	Pso-ED 10.9 (5–24.6) Pso + ED 10.3 (5–26.9)	Pso-ED 7.8 (1–30) Pso + ED 15.5 (1–50)	none	FMD, NMD (vessel dilatation as the % of baseline value, %)/not specified (occlusion)	BA	↓ FMD (*p* < 0.05)	↑ YKL-40 (*p* < 0.05) ↑ CRP (*p* < 0.05) Pso + ED vs. controls + ED: ↑ YKL-40 (*p* < 0.05)
**Haberka, 2018,** [226]	Poland	Case-control Study	80	39	Mild to moderate	18.6 ± 10.5	15.3 ± 11.2	none	cfPWV (m/s), no occlusion site, FMD (vessel dilatation as the % of baseline value, %)/proximal portion of the arm (occlusion) cIMT (mm), no occlusion site	cfPWV—sensors (CCA and CFA) FMD- BA, above the antecubital fossa cIMT—CCA	↑ cIMT(mm) (*p* < 0.05) ↓ FMD% (*p* < 0.001) ↔ PWV m/s	↑ AOPPs (*p* < 0.001), sign. assoc. with IMT (r = 0.3), FMD (r = -0.25) ↑ visfatin (*p* < 0.001) ↔ osteoprogerin, ↔ nesfatin
**Holzer, 2021,** [227]	Austria	Randomized Controlled Trial	65		Moderate to severe/PASI ≥ 10	Adalimumab group: 16.3 ± 5.8 FAE group:16.4 ± 5.9	Adalimumab group: 11.9 ± 11.3 FAE group:10.1 ± 8.8	Intervention with: Adalimumab (*n* = 33, 50.8%) FAE (*n* = 32, 49.2%) + NB-UVB (for non-responders)	FMD, NMD (vessel dilatation as the % of baseline value, %)/not specified (occlusion) cIMT (mm), no occlusion site	FMD—BA, above the antecubital fossa cIMT—1st cm of the CCA	Adalimumab group: ↑ FMD% after intervention (*p* = 0.048) FAE group: ↔FMD% Both groups: ↔ NMD%, ↔cIMT (mm)	Adalimumab a.i.:↓hsCRP (*p* = 0.022); FAE a.i.: ↔ hsCRP; ↓ *p*-selectin (*p* = 0.034) Both groups a.i.: ↓E-selectin (FAE: *p* = 0.041; adalimumab: *p* = 0.001)
**Erturan, 2014,** [228]	Turkey	Case-control Study	56	53	Mild to moderate/PASI 0.1–49.9	3 (range 0.6–27)	5.5 (range 0.5–50)	None (at least 3 months prior)	FMD (vessel dilatation as the % of baseline value, %)/forearm, bottom of the cuff on the wrist (occlusion) IMT(mm), no occlusion site	FMD—BA, 2–5 cm proximal to the antecubital fossa cIMT—previous segment of the bifurcation of the CA	↓ FMD % (*p* = 0.0001) ↔ cIMT	↑ sCD40L (*p* = 0.012) ↔ homocysteine ↔ ESR ↔ hsCRP
**Karadag, 2010,** [204]	Turkey	Case-control Study	75 (24 + PsA)	50	All PASI included	4.4 (1.8–34)	No data provided	No data obtained	FMD (vessel dilatation as the % of baseline value, %)/proximal forearm (occlusion)	BA	Pso vs. controls: ↓ FMD% (*p* < 0.001), no correlation with PASI PsA vs. Pso: ↓ FMD (*p* = 0.096)	↑ ESR (*p* = 0.006)
**Białecka, 2021,** [229]	Poland	Case-control Study	62 (6 + PsA)	42	All PASI included	14.92 ± 6.99	Assessed, data not provided	Data obtained on past use of systemic therapy (systemic treatment was used in *n* = 39; 62.9%)	cIMT (mm), no occlusion site cardiac CT: calcium score according to the Agatston scale (CS); mass of calcifications (CM, mg); the volume of calcifications in coronary arteries (CV, mm^3^)	cIMT—Both CCA, 2 cm from their bifurcation	↑ cIMT (*p* < 0.0001); no correlation with PASI or CRP ↑ amount of calcification	↑ CRP (*p* < 0.0001)
**Bańska-Kisiel, 2016,** [230]	Poland	Cross-sectional Study	74	none	Mild to moderate/PASI ≤ 50	18.7 ± 10.6	17.1 ± 11.2	Biologics (*n* = 5; 7%)	cIMT (mm), no occlusion site	Both CCA, distal segments	Association between cIMT and PASI (r = 0.33; *p* = 0.007)	/
**Troitzsch, 2012,** [231]	Germany	Cross-sectional Study	72	1955	No data	No data	No data	No data provided	cIMT (mm), no occlusion site	Both CCA (10 consecutive measurement points, in 1 mm steps, from the bulb of both sides)	↑ cIMT (*p* = 0.001) ↔ carotid plaque prevalence	↑ hsCRP (*p* = 0.003)
**de Oliveira, 2019,** [232]	Brazil	Case-control Study	11	33	Severe/PASI > 10	No data	No data	MTX (*n* = 2, 18%)	PWV (m/s), AIx, arm (occlusion) cIMT(mm), no occlusion site	PWV—not specified cIMT—1 cm from the posterior wall of the CCA	↑ PWV (*p* = 0.033) ↑ IMT (left CCA) above the 75th centile (*p* = 0.045)	↑ CRP (*p* < 0.001)
**Fabi, 2022,** [233]	Italy	Case-control Study	20 * age < 18	20	Not specified	2.64 ± 2.6	1.84 ± 1.18	Cyclosporine (*n* = 3, 15%), 2 switched to guselkumab	cIMT(mm), no occlusion site	Both CCA, at least 5 mm below its end	↑ cIMT (right, *p* = 0.001; left, *p* = 0.00), positively correlating with disease duration	/
**Awad, 2017,** [234]	Egypt	Case-control Study	45	45	Not specified/all PASI included	10.18 ± 4.6	cIMT < 1 mm: 10.27 ± 14.07 cIMT > 1 mm: 11.33 ± 6.98	none	cIMT(mm), no occlusion site	Both CCA, distal portion of the CCA (10–20 mm proximal to the carotid bulb)	↑ cIMT (*p* < 0.001), positively correlating with PASI (r = 0.78, *p* < 0.001), serum psoriasin (r = 0.48, *p* > 0.01) and serum koebnerisin (r = 0.48, *p* < 0.01), but not with disease duration	↑ psoriasin (*p* < 0.001) ↑ koebnerisin (*p* = 0.001), higher levels in patients with subclinical atherosclerosis (*p* = 0.04)
**Liu, 2015,** [235]	China	Case-control Study	35	20	BSA > 10%	15.5 ± 12.7	14.0 ± 7.2	MTX (*n* = 13, 37.1%) Retinoids (*n* = 2, 5.7%)	haPWV (m/s), no occlusion site, cIMT(mm), no occlusion site AI	haPWV—precordium and both posterior PA cIMT—max. thickness point along a 1-cm section of the CCA proximal to the carotid bulb, both sides	CD34 + EPC was independently predictive of increased haPWV	↓ CD34 + EPC (*p* = 0.02); neg. correlating with haPWV (r = -0.43, *p* = 0.01) ↔ CD34/KDR + EPC, ↔ CD133/KDR + EPC and ↔ CD133 + EPC
**El-Mongy, 2009,** [236]	Egypt	Case-control Study	80 (25 + PsA)	50	Not specified/all PASI included	29.1 ± 16	12.6 ± 9.5	No data provided (patients treated with cyclosporine or retinoid were excluded)	cIMT(mm), no occlusion site	right CCA, 1 cm distal to the carotid bifurcation in the posterior wall	↑ cIMT (*p* < 0.001), positively correlating with age (r = 0.6, *p* ≤ 0.001), duration of the disease (r = 0.4, *p* = 0.001) and PASI (r= 0.5, *p* ≤ 0.001)	↑ CRP (*p* ≤ 0.001) ↑ ESR (*p* = 0.004)
**Martinez-Lopez, 2018,** [237]	Spain	Prospective Cohort Study	53 (21 + PsA)	Self-controlled, 8 m	PASI ≥ 5	9.46 ± 3.62	17.33 ± 10.78	Systemic therapy (*n* = 30, 56.6%) Cyclosporine (*n* = 10, 18.8%), MTX (*n* = 10, 18.8%), acitretin (*n* = 10, 18.8%); Biologics (*n* = 23, 43.4%); TNF-α inhibitor (etanercept, infliximab, adalimumab), (*n* = 13, 24.5%); anti-IL12/23 (ustekinumab), (*n* = 10, 18.8%) Wash out period of 3 months before baseline	cIMT (mm), no occlusion site	cIMT—left CCA, 1 cm from the carotid bifurcation (6 measurements)	All patients: Decreasing tendency IMT (*p* = 0.086) MTX a.i.: ↓ IMT (*p* = 0.045) ustekinumab a.i.: ↓ IMT (*p* = 0.010)	/
**Piros, 2021,** [238]	Hungary	Prospective Cohort Study	31 (17 + PsA)	Self-controlled, 6 m	Severe psoriasis/PASI > 10	18 (14–24)	24 (16–28)	anti-IL-17 therapy- intervention: secukinumab (*n* = 20, 64.5%), ixekizumab (*n* = 11, 35.5%)	cIMT (mm) bIMT (mm) fIMT (mm); no occlusion site	cIMT—CCA; bIMT—middle third of the BA fIMT—middle third of the CFA * on both sides	6 months after baseline, a.i. ↓ cIMT, ↓ bIMT, ↓ fIMT (*p* < 0.001 for all)—the improvement was more significant in non-calcified arteries than in calcified arteries	/
**Jokai, 2013,** [239]	Hungary	Prospective Cohort Study	16	Self-controlled, 6 m	Severe psoriasis/PASI > 15	Baseline: 25.64 (21.2–32.4); ↓ of PASI after 6 months for 1.04 (0–8.8)	16.8 (4–40)	No biologic therapy at baseline; intervention with TNF-α inhibitors: etanercept (*n* = 3, 18.8%), infliximab (*n* = 7, 43.8%), adalimumab (*n* = 6, 37.5%) during 6 months	cIMT (mm), bIMT (mm), no occlusion site	cIMT—carotid bifurcation bIMT—middle third of the BA	Group 1— no apparent atherosclerosis (*n* = 13) ↓ after intervention cIMT(mm) (*p* = 0.011) ↓ after intervention bIMT(mm) (*p* = 0.006) Group 2—atherosclerosis present (*n* = 3) ↔ cIMT, ↔ bIMT (but increasing tendency)	/
**Ikonomidis, 2015,** [240]	Greece	Case-control Study	59	59 CAD patients; 40 healthy controls	All PASI included	11.5 ± 8	5.1 ± 1.25	Ciclosporine (*n* = 59, 100%)	cfPWV (m/s), augmentation index (CAI, %), no occlusion site, FMD (vessel dilatation as the % of baseline value, %)/occlusion site not specified cIMT(mm), no occlusion site CFR (ratio of peak diastolic velocity after adenosine infusion to peak diastolic velocity at rest), no occlusion site	cfPWV—sensors (CCA and CFA FMD—BA cIMT—CCA, bulb, ICA; on both sides CFR—color Doppler on LAD	Compared to healthy controls: ↑ cfPWV; ↑ CAI, ↑ IMT (*p* < 0.05 for all); IMT values correlating with PASI (r = 0.67, *p* < 0.01) ↓ FMD, ↓ CFR Compared to CAD patients: ↔ cfPWV; ↔ CAI, ↔ IMT ↔ FMD, ↔ CFR	Compared to healthy controls: ↑ MDA, ↑ IL-6 (*p* < 0.05 for both), correlating with cIMT (r = 0.35, *p* = 0.01 and r = 0.58, *p* < 0.001) Compared to CAD patients: ↔ MDA, ↔ IL-6
**Robati, 2014,** [241]	Iran	Case-control Study	60	60	All PASI included	23.45 (14.92–33.18)	10 (4–16.5)	None (exclusion criteria was systemic therapy within the last 6 months)	cIMT (mm), no occlusion site	Right CCA, 1 cm proximal to the bifurcation (at least 3 measurements)	↑ cIMT (*p* < 0.0001)	↑ leptin, ↑ resistin (*p* < 0.0001)
**Antonucci, 2014,** [242]	Italy	Case-control Study	40	40	Moderate to severe/PASI > 10	16.1 ± ?	Not assessed	Exclusion criteria were: cyclosporine, oral retinoids, systemic steroids; no other data on therapy available	cIMT (mm), no occlusion site	cIMT—CCA, 1 cm proximal to the bifurcation	↑ IMT (*p* < 0.001), positively correlating with PASI (r = 0.515, *p* < 0.01), not with BMI	/
**Marovt, 2020,** [243]	Slovenia	Prospective Cohort Study	15 (4 + PsA)	Self-controlled	Moderate to severe/PASI > 10	PASI 16.78 (11.0–19.8) BSA 12.62 (8–20)	20.9 (range 3–52)	Intervention with anti-IL-23/IL-17: ustekinumab (*n* = 4, 26.67%); secukinumab (*n* = 10, 66.67%); ixekizumab (*n* = 1, 6.67%)	cfPWV (m/s), no occlusion site, aortic AIx, cIMT (mm), no occlusion site	CfPWV—CA, FA cIMT—CA, bifurcation level, both sides	↔ cfPWV ↔ cIMT ↑ central aortic diastolic pressure (mmHg) (*p* = 0.03)	/
**Elsheikh, 2013,** [244]	Egypt	Case-control Study	60	20	Mild, moderate, severe/all PASI included	18.49 ± 11.29	11.25 ± 6.95	None (at least 6 weeks prior to cIMT)	cIMT (mm; internal diameter—ID; arterial wall mass index—AWMI), no occlusion site	Both sides at three points: - CCA (10 mm before the bulb) - Bulb (5–10 mm cranially to the start of the bulb - Internal carotid artery column after the flow divider	↑ cIMT (*p* = 0.001); ↑ AWMI (*p* = 0.010) ↓ ID (*p* = 0.001) - independent predictor of cIMT: duration of disease (r = 0.425, *p* = 0.008); age (r = 0.362, *p* = 0.021), PASI score (r = 0.326, *p* = 0.014); BMI (r = 0.243, *p* = 0.019)	/
**Yiu, 2010,** [206]	China	Case-control Study	52	50	BSA > 10	14.7 ± 12.1	15.4 ± 7.1	Methotrexate (*n* = 26, 50%)	baPWV (m/s), no occlusion, PAT (index), proximal forearm of the studied hand (occlusion)	baPWV—ATP and BA; PAT—tip of both middle fingers	Psoriasis vs. controls: ↑ baPWV (*p* < 0.01) ↔ PAT index Psoriasis patients on MTX vs. without MTX: ↔ baPWV ↔ PAT index no correlation between baPWV and PAT index (r = 0.09, *p* = 0.40)	↑ hsCRP (*p* < 0.01)—correlating with baPWV (r = 0.51, *p* < 0.01) and with PASI (r = 0.48, *p* < 0.01)
**Kim, 2015,** [245]	South Korea	Case-control Study	54	60	Mild and moderate to severe/all PASI included	10.7 + 7.0	10.4 + 9.7	Data on previous systemic treatment obtained: *n* = 49, 90.7% had received systemic treatment at some point	BSI (β) cIMT (mm), no occlusion site	BSI—region 2 cm from the carotid bifurcation toward the center of the body cIMT—1 cm distal to the far wall of each CCA	↑ BSI (*p* < 0.001), correlating with PASI ↔ cIMT (intended to be ↑, no significance)	/
**Patschan, 2018,** [246]	Germany	Case-control Study	30	26	Not specified	10.2 ± 2.0	18.3 ± 2.7	Past/present treatment with biological drug (*n* = 10, 33%)	cfPWV (m/s), augmentation index, AI; no occlusion site	Sensors on CA and FA	↔ PWV (m/s)	↑CRP ↔ CD133+/KDR+ (EPC) cells
**Pina, 2016,** [219]	Spain	Prospective Cohort Study	29	Self-controlled	Moderate to severe psoriasis	18.9 ± 7.8	18.2 ± 12.1	Anti-TNF-α (intervention): adalimumab Washout period from other systemic therapies of 4 weeks	FMD (vessel dilatation as the % of baseline value, %), forearm (occlusion); PWV	FMD—BA, 2–12 cm proximal to the antecubital fossa PWV—right CCA	A.i. vs. baseline: ↑ FMD%(*p* = 0.008) ↓ PWV (*p* = 0.03)	hsCRP?
**Balta, 2014,** [247]	Turkey	Case-control Study	32	35	All PASI included	Assessed, but values not presented in paper	Assessed, but values not presented in paper	No data provided	PWV (m/s), augmentation index (AIx), BA (occlusion)	Distance between jugular notch and symphysis pubis	↑ PWV (m/s), (*p* = 0.01), no correlation with disease duration or PASI	↑ hsCRP (*p* = 0.01)
**Dregan, 2018,** [248]	United Kingdom	Cross-sectional Study	2091	165 149	Presence of psoriasis diagnosis/all included	Not assessed	Not assessed	Corticosteroids (*n* = 166, 8% DMARDs (*n* = 168, 8%)	photoplethysmography (arterial stiffness index, SI, m/s), no occlusion	Index finger of the dominant hand	↑ SI (*p* = 0.016)	/
**Choi, 2016,** [249]	South Korea	Case-control Study	103	103	All PASI included	8.7 + 5.5	3 (0.5–10)	No data provided	CAVI, right brachial, right ankle (occlusion) PWV(m/s), cAIx	PWV—between aortic valve and ankle cAIx—BA, RA	↑ CAVI (*p* = 0.03) cAIx, correlating with disease duration (r = 0.319, *p* = 0.001), not with PASI	↑ CRP (*p* = 0.025)
**Hansen, 2018,** [250]	Denmark	Cross-sectional Study	254	4431	Self-reported psoriasis/all included	Not assessed	Not assessed	Not assessed	photoplethysmography (arterial stiffness index, SI), no occlusion	Index finger of the non-dominant hand	↑ SI (*p* = 0.04)	↑ hsCRP
**Jensen, 2014,** [251]	Denmark	Randomized Controlled Trial	30 Pso, low energy diet	30 Pso, normal diet	All PASI included	4.8 (3.8–8.2) intervention group; 5.5 (3.6–6.8) controls	Not assessed	Not assessed	PAT (reactive hyperemia index, RHI)/Brachial artery (occlusion on the upper arm)	Both index fingers	↔ RHI	↔ hsCRP ↔ VCAM ↔ ICAM
**Nakao, 2018,** [222]	Japan	Cohort Study	15 (7 + PsA)	Self-controlled	Not specified	5.7 (3.2–12.8)	Mean 18.7	Intervention with anti TNF-α: infliximab	RH-PAT (RHI), arm opposite to the dominant arm (occlusion)	Fingers of each hand	6 weeks: ↔ RHI in responders ↓ trend RHI in non-responders (*p* = 0.09)	↓ CRP, ↓ ESR (*p* = 0.016)
**Sunbul, 2015,** [252]	Turkey	Case-control Study	50	50	Not specified	13.7 ± 8.9	13.5 ± 10.7	No data (data obtained about previous medication)	PWV(m/s), AIx		↑ PWV (*p* = 0.001) ↑ AIx (*p* = 0.001), no correlation with PASI observed	↑ NLR (*p* = 0.002)
**Altekin, 2012,** [253]	Turkey	Case-control Study	57	60	Not specified/all PASI included	7.8 ± 7.4	11.3 ± 8.5	None (no systemic immunosuppressive therapy at least 6 months prior)	cfPWV(m/s), cIMT (mm), no occlusion site	cfPWV—CA, FA cIMT—1 cm segment of both CCA, 2–3 cm distal to the bulb	↑ cfPWV (*p* < 0.001), positively correlating with PASI (r = 0.417, *p* = 0.001) ↑ max cIMT (*p* < 0.001) ↑ mean cIMT (*p* < 0.001)	/
**Enany, 2011,** [254]	Egypt	Case-control Study	50	10	All PASI included	20.99 ± 16.67	6.50 ± 2.95	None (at least 6 months prior)	cIMT(mm), no occlusion site	CCA, 1 cm proximal to the carotid bulb; bulb; ICA, 1 cm distal to the carotid bifurcation	↑ cIMT (*p* < 0.05), correlating with age, disease duration, BMI, PASI score, systolic blood pressure, diastolic blood pressure, leptin levels, LDL levels and triglyceride levels	↑ leptin (*p* < 0.05)
**Divito, 2018,** [255]	Italy	Case-control Study	↓ CV R 34; ↑ CVR 23	↓ CVR 39; ↑ CVR15	Severe/PASI not specified	No data provided	No data provided	No data provided	PWV (m/s)	Not specified	Low CV risk, Pso vs. controls: ↔ PWV High CV risk, Pso vs. controls: ↑ PWV (*p* = 0.037)	/
**Asha, 2014,** [256]	India	Case-control Study	80	80	Not specified/all PASI included	15.60 ± 10.79	3.42 ± 2.56	No data provided	cIMT(mm), no occlusion site	Both CA	↑ mean cIMT (*p* < 0.001), significant cumulative association with leptin and apoB/apoA-I	↑ leptin (*p* < 0.001)
**Usta, 2011,** [203]	Turkey	Case-control Study	29	25	Not specified/all PASI included	4.6 ± 3.8	13 ± 10	None (at least 1 month prior)	FMD, NMD (vessel dilatation as the % of baseline value, %), upper arm proximal to the imaged artery segment (occlusion);	BA, 2–4 cm above the antecubital fossa	↔ FMD ↔ NMD	↑ CRP (*p* = 0.011) ↑ fibrinogen (*p* = 0.011) ↔ ADMA
**Arias-Santiago, 2012,** [257]	Spain	Case-control Study	72 (25 +PsA)	61	Severe/PASI > 10	mean 19.25	mean 17.64	None (at least 2 months prior)	cIMT(mm), no occlusion site	Distal portion of the both CCA, 1 to 2 cm proximal to the carotid bulb	↑ right cIMT (*p* = 0.013) ↑ left cIMT (*p* = 0.042) ↑ presence of carotid atheroma plaques (*p* < 0.001)	↑ fibrinogen, ↑ CRP, ↑ ESR, ↑ D-dimer, ↑ homocysteine
**Alba, 2018,** [210]	USA	Case-control Study	9 (1+ PsA)	9	≥5% BSA	16 ± 2 BSA	No data provided	None	LDF (NO-dependent vasodilatation—ΔCVC_local heating_ and CVC_post-l-NAME_ SNP-induced vasodilatation (flux/mmHg); vascular adrenergic responsiveness, logEC50)	Cutaneous microcirculation, forearm skin	↓ NO-dependent vasodilation (*p* < 0.01), correlating with BSA (r = 0.54, *p* = 0.04) ↔ SNP-induced vasodilatation After ascorbate infusion: ↔ NO-dependent vasodilation ↔ vascular adrenergic responsiveness ↔ max NE-induced vasoconstriction	/

Legend. PAT—digital peripheral artery tomography; FMD—flow-mediated dilatation; NMD—nitroglycerin-mediated dilatation; cfPWV—carotid-femoral pulse wave velocity; crPWV—carotid-radial pulse wave velocity; cIMT—carotid intima-media thickness; bIMT—brachial intima-media thickness; CFR—coronary flow reserve, by doppler echocardiography; baPWV—brachial-ankle pulse wave velocity; haPWV—heart to ankle pulse wave velocity; PWA—pulse wave analysis; CAVI—Cardio-Ankle Vascular Index; cAIx—central augmentation index; RH-PAT—reactive hyperemia-peripheral arterial tonometry; LDF—Laser Doppler flowmetry; CCA—common carotid artery; FA—femoral artery, BA—brachial artery; CFA—common femoral artery; RA—radial artery; ICA—internal carotid artery; LAD—left anterior descending; CFA—common femoral artery; ATP—posterior tibial artery; FAE—fumaric acid esters; a.i.—after intervention; PsA—psoriatic arthritis; CT—computed tomography; MTX—methotrexate; BSI—beta stiffness index; AI/AIx—augmentation index; ESR—erythrocyte sedimentation rate; ED—endothelial dysfunction; CVR—cardiovascular risk; NLR—neutrophil-to-lymphocyte ratio; PBR—perfused boundary region, a marker of glycocalyx barrier function; PA—popliteal artery; CVC—cutaneous vascular conductance; m- months; β—standard regression coefficient; ↔, no significant difference between groups; ↑, increased; ↓, decreased; *—special remark.

## 7. Increased CVD Risk in PV Patients Due to Impaired Endothelial Function

In the past decades, it has become clear that psoriasis, as a systemic inflammatory condition, may be accompanied by various systemic comorbidities, including psoriatic arthritis, cardiovascular and cerebrovascular diseases, hyperlipidemia, hypertension, obesity, diabetes and psychiatric disorders like depression and anxiety [258,259]. Among the comorbid conditions associated with psoriasis, cardiovascular diseases are of particular importance as they contribute to higher mortality rates of psoriasis patients compared to controls [191,260,261,262]. The incidence of both myocardial infarction (MI) and cerebrovascular accident (CVA) has been reported to be increased in psoriasis patients, correlating with the duration of this autoimmune disease, as well as clinical severity assessed by PASI, body surface area (BSA) and the need for systemic therapy or phototherapy for psoriasis [263,264,265,266,267,268,269,270,271]. Data from previous studies suggest that psoriasis patients die approximately 6 years earlier than non-affected individuals and that cardiovascular morbidities are the most prominent individual cause of death in those patients [260]. Furthermore, the presence of cardiovascular diseases in psoriasis patients has been linked to increased healthcare utilization, as well as higher healthcare costs needed to manage such conditions [258], making this also a financial burden for public health system.

Even though ample evidence from multiple studies shows that psoriasis patients have a higher burden of the so-called traditional CV risk factors, such as cigarette smoking, obesity, elevated blood pressure, insulin resistance or type II diabetes mellitus, and hyperlipidemia [272,273,274], the majority of available research suggests that psoriasis represents an independent cardiovascular risk factor. For example, in young psoriasis patients who had a low Framingham Risk Score (FRS) when tested for risk of developing cardiovascular events by obtaining information on traditional risk factors, vascular inflammation measured by ^18^F-fluorodeoxyglucose positron emission tomography computed tomography (18-FDG PET/CT), which appears to have prognostic value for future CV events, was increased and associated with longer disease duration [267,275,276,277]. Thus, it appears that prolonged exposure to these inflammatory conditions may contribute to increased cardiovascular risk in psoriasis patients [278]. However, as regards the traditional risk factors for the development of CVDs, it should be noted that psoriasis seems to increase the risk of their development. For example, psoriasis increases the incidence of both diabetes and dyslipidemia, meaning that this autoimmune disease may also contribute to the increased overall cardiovascular risk by affecting the incidence of the traditional risk factors [102].

Moreover, it has been suggested that even in mild-to-moderate psoriasis there is a greater risk to develop severe cardiovascular events, since elevated carotid intima-media thickness (IMT), decreased flow mediated dilation (FMD) and greater levels of advanced oxidative protein products (AOPPs) have all been reported in psoriasis patients compared to non-affected individuals; but no difference in pulse wave velocity (PWV) has been observed. However, since the upper threshold for moderate psoriasis in this report was defined as PASI score of 50 and the mean PASI was above 18 [226], which is usually considered as severe psoriasis [279], it would be of value to test these conclusions in reference to mild-to-moderate psoriasis in further studies. However, in a study where mild-to-moderate psoriasis was defined as PASI < 10, there was no difference found in arterial stiffness measured by PWV, and no other assessment techniques for endothelial function were used [208]. Even though PASI is a widely used instrument for assessing psoriasis severity, it has some limitations. For example, in a chronic recurrent disease such as plaque psoriasis, which is characterized by exacerbations and remissions, a one-off measurement such as PASI does not provide information on long-term inflammation or disease severity. Therefore, PASI might not be the ideal indicator of the severity of disease progression in psoriasis [280,281]. 

Furthermore, two different meta-analyses confirmed that there is a higher relative cardiovascular risk, particularly in younger individuals suffering from psoriasis, and in those with more severe clinical presentation [282,283]. It has been suggested that the reason for an elevated CV risk in psoriasis patients lies in the endothelial dysfunction induced by chronic inflammation, observed in several studies on patients with psoriasis [201,202,207,210,284,285], in which TNF-α and oxidative stress seem to play important roles [119], as already mentioned in the previous section. In fact, it has been found that endothelium-dependent vasodilatation is ameliorated in patients with psoriasis [201,202,207,284], while the vasodilation induced by sodium nitroprusside (SNP) appears to be intact [201,202]. 

The importance of endothelial dysfunction also lies in the fact that this is a quantifiable condition reflecting early reversible damage to vascular homeostasis [286], preceding the formation of atherosclerotic plaques, contributing to the progression in the plaque formation and thus leading to the occurrence of cardiovascular events [44,45,46,286,287]. In endothelial dysfunction we can observe the loss of antithrombotic properties of the endothelium, along with the upregulation of adhesion molecules, like VCAM-1, ICAM-1, which enable leukocyte adhesion, i.e., support the priming of monocytes to the vascular wall, which are then further differentiated into foam cells after the accumulation of modified lipoproteins, which is a crucial step in the process of atherogenesis [46,47,48,49,288,289,290].

In a report from a prospective cohort study, flow-mediated dilation (FMD), a measurement technique for endothelial dysfunction, negatively correlated with cardiovascular events independently of other, traditional CV risk factors [291], underlining the potential prognostic value of endothelial dysfunction. Due to the fact that endothelial dysfunction is reversible, it might be a promising and valuable therapeutic objective in the primary prevention of future major cardiovascular events in psoriasis patients [195]. 

## 8. Rationale for Reducing Dietary Salt Intake and Antioxidant Supplementation in Patients Suffering from Psoriasis

Sodium is an electrolyte crucial for numerous processes in the organism and is therefore considered to be an essential nutrient, necessarily present in small quantities in the human diet. However, in most Western and Asian countries, the dietary intake of sodium is markedly surpassing the maximum daily sodium intake of 2000 mg (i.e., 5 g salt) recommended by the World Health Organization (WHO) guidelines established to prevent diet-related non-communicable diseases [292,293,294,295]. As already mentioned in this paper, increased dietary salt intake leads to dysregulation of the immune system through promoting the differentiation of CD4+ cells to Th17 cells, and therefore supporting the development and maintenance of inflammation in autoimmune diseases [19,296,297,298]. Also, an emerging body of evidence supports the thesis that HS intake disturbs the redox balance in the direction of oxidative stress, which also seems to largely participate in this process [52]. 

Diet and nutrition are generally considered to be a modifiable risk factor in the occurrence of many diseases, including autoimmune diseases [299]. The identification of food and/or nutritional supplements affecting the disease course in terms of either improving the disease or leading to its further exacerbation, can be of potential therapeutical importance. It has been previously reported that psoriasis patients’ diet has an impact on the disease course [300,301]; e.g., diet with low-calorie intake and diet enriched with *n*-3 polyunsaturated fatty acids (PUFAs) resulted in clinical improvement in patients suffering from psoriasis [302,303,304,305]. Even though it seems that diet has an impact on the disease, at this point there are no official dietary guidelines for patients with psoriasis. 

As previously stated, while the serum sodium level is regulated to be closely maintained at around 140 mmol/L [306], it has been reported that the excess sodium can either be excreted from the body or accumulated in body tissues such as the skin [16,161,162,163,164]. In a recent study by Maifeld et al., it has been observed that patients with PASI > 5 have a greater sodium content in both lesional and nonlesional skin compared to patients with milder psoriasis and to healthy controls [16]. However, dietary sodium intake has not been tested in this study, so the impact of dietary intake on the accumulation of sodium in the skin of psoriasis patients still remains to be explained [167]. Since it is known that HS conditions shift the differentiation of CD4+ cells towards Th17 cells [19,296,297,298], disturb Treg cells [307], and also lead to an increase in ROS production, lowering of salt intake could have therapeutical implications in the context of psoriasis and associated comorbidities, including the damage of endothelial function [52,53], by a decrease of inflammation and oxidative stress [167]. 

Since LS diet can lead to the recovery of endothelial microvascular function in both normotensive and hypertensive individuals [308,309], the same effect could be expected in psoriasis patients with established endothelial dysfunction [201,202,207,210,284,285]. For example, an in vitro study conducted by Wild et al., has shown that the effect of TNF-α on the activation of endothelial cells under non-uniform shear stress is increased in HS conditions and that this effect is dependent on sodium concentrations [67]. This provides an insight into the potential of LS dietary intake to influence one of the key mechanisms by which microvascular function is disrupted in psoriasis patients. Since TNF-α plays a role in the immunopathogenesis of psoriasis and is therefore elevated in psoriasis patients [310], a change in dietary habits of these patients could make a greater difference in endothelial function recovery than in healthy individuals. All this leads to the assumption that a LS diet might benefit psoriasis patients. 

LS intake could lead to the recovery of endothelial dysfunction, which is the first, but still reversible, step in the process of atherogenesis [195]. This could further lead to a decrease in CVD risk, which is present in psoriasis patients independently of other, traditional, CV risk factors, and could decrease morbidity, mortality and public health expenses linked to cardiovascular comorbid conditions in psoriasis patients. It would, indeed, be of great value to further examine the effect of LS diet on the endothelial function in patients with psoriasis in vivo. 

In addition, it has been shown that psoriasis patients have an unhealthy diet characterized by consuming more simple carbohydrates, total fat and n-6 PUFA, with less fibers, proteins, complex carbohydrates, fruits, vegetables and n-3 PUFA when compared to healthy controls [311,312], which could contribute to the pro-inflammatory state seen in psoriasis [311,313,314]. The OR for psoriasis has been lower in participants consuming more fruit and vegetables [314]. Moreover, vegetarian diets have been reported to have a beneficial effect on disease severity in psoriasis patients [315,316]. This effect of dietary intake of fresh fruit and vegetables might be due to the high content of exogenous antioxidants in this type of food, such as vitamin C, flavonoids and carotenoids, which might increase antioxidative capacity in psoriasis patients. Another potential mechanism could be the lower intake of arachidonic acid in vegetarian diets, which could decrease the level of its proinflammatory products [314,317]. 

Vitamin E (α tocopherol) is a liposoluble antioxidant vitamin, known to prevent lipid peroxidation [318,319], which is a process observed in patients with psoriasis [79,97]. Also, vitamin E appears to play a role in skin barrier maintenance by preventing the peroxidation of lipids in the corneal skin layer [320]. Importantly, one meta-analysis reported low levels of vitamin E in serum of psoriasis patients [321], while some researchers found that vitamin E levels negatively correlated with disease severity [322]. In a randomized double-blind clinical trial with patients with psoriatic arthritis and erythroderma, supplementation with an antioxidant combination of vitamin E, selenium and coenzyme Q10 yielded clinical improvement, and it decreased the measured oxidation markers [323]. Furthermore, in a study on young healthy individuals on a short-term HS diet, the supplementation with vitamin C and vitamin E prevented the decrease in endothelium-dependent vasodilatation caused by the HS diet [53]. In future studies, it might be useful to investigate whether this effect of vitamins C and E on microvascular function is present in psoriasis patients, especially in the settings of HS dietary loading. 

Since selenium is an essential component of selenoproteins, such as glutathione peroxidase (an important cellular antioxidant enzyme), its nutritional supplementation has been studied in psoriasis patients in a few studies [324]. In addition to its antioxidant role, selenium has been shown to reduce TNF-α, a cytokine important in psoriasis. However, in the studies conducted so far, no change in disease severity has been observed in trials with psoriasis patients after the supplementation of selenium [325,326].

In addition, it has already been mentioned that the intake of n-3 PUFA, i.e., docosahexaenoic acid (DHA) and eicosapentaenoic acid (EPA), which also have antioxidant properties, is lower in patients with psoriasis than in controls [311,312,327]. Furthermore, a positive correlation between PASI and n-6/n3 ratio has been demonstrated, as well as a significant negative correlation between PASI and both EPA and DHA levels [328]. Thus, supplementation with n-3 PUFA has been studied in several studies on patients with psoriasis and many of those studies found that additional intake of n-3 PUFA has a beneficial effect on psoriasis [304,329,330,331]. Furthermore, the intake of n-3 PUFA-enriched hen eggs improved endothelium-dependent vasodilatation in healthy individuals [332], which might also be beneficial for psoriasis patients, who have impaired microvascular function. 

## Data Availability

The data presented in this study are available in this manuscript.
